# Effects of statins on plaque characteristics of intracranial atherosclerosis assessed by high-resolution magnetic resonance imaging

**DOI:** 10.3389/fneur.2026.1724878

**Published:** 2026-01-28

**Authors:** Hongshan Chu, Shibo Dong, Hongyu Hao, Ruisheng Duan

**Affiliations:** 1 Department of Neurology, Hebei General Hospital, Shijiazhuang, Hebei, China; 2 Department of Medical Imaging, Hebei General Hospital, Shijiazhuang, Hebei, China

**Keywords:** enhancement, high-resolution magnetic resonance imaging (HR-MRI), ischemic stroke, middle cerebral artery, statin

## Abstract

**Objective:**

To investigate clinical factors associated with unstable intracranial plaques and examine the relationship between pre-stroke statin use and plaque instability using high-resolution magnetic resonance imaging (HR-MRI).

**Methods:**

In this retrospective cross-sectional study, we enrolled 116 patients with acute anterior circulation cerebral infarction (within 7 days of onset) due to symptomatic intracranial atherosclerosis, all of whom underwent HR-MRI during hospitalization. Based on pre-stroke statin exposure, patients were grouped into a no-statin group and a statin-treatment group; based on culprit-plaque enhancement, they were further divided into enhancement and non-enhancement groups. Using HR-MRI, we systematically evaluated vascular morphometrics of the culprit artery (vessel area, lumen area, degree of stenosis, and remodeling index) and plaque activity parameters (enhancement grade).

**Results:**

Eighteen patients (15.5%) had used statins prior to stroke onset. Compared with the no-statin group, the statin group had significantly lower total cholesterol (TC), low-density lipoprotein cholesterol (LDL-C), and non–high-density lipoprotein cholesterol (non-HDL-C) (*p* = 0.001, *p* < 0.001, *p* < 0.001). Infarct-pattern distributions differed between groups (*p* = 0.023): in the statin group, deep-only infarcts (50.0%) and cortical-only infarcts (33.3%) were more frequent, whereas large cortical/cortical–deep infarcts predominated in the no-statin group (50.0%). Plaque enhancement was less frequent in the statin group (*p* = 0.015) multivariable logistic regression, identified body mass index (BMI) (*p* = 0.021; OR = 1. 157; 95% CI: 1.023–1.309) and lack of statin use (*p* = 0.028; OR = 3.351; 95% CI: 1.143–9.823) as independent factors associated with plaque enhancement.

**Conclusion:**

Pre-stroke statin therapy stabilizes intracranial plaques by lowering lipids and suppressing plaque enhancement. It independently protects against enhancement and is associated with fewer large cortical infarctions, whereas elevated BMI is an independent risk factor for enhancement.

## 1 Introduction

Intracranial atherosclerosis (ICAS) is a major cause of ischemic stroke and is particularly prevalent in Asian populations (1). Rupture of unstable atherosclerotic plaques can precipitate severe cerebrovascular events, including cerebral infarction and transient ischemic attack (TIA) (2). Accurate characterization of plaque features is therefore essential for risk stratification and stroke prevention. Conventional angiographic methods—computed tomography angiography (CTA) and magnetic resonance angiography (MRA)—primarily evaluate luminal stenosis. However, growing evidence indicates that plaque instability, determined by morphology and composition, may better predict ischemic events than stenosis severity alone (3). High-resolution magnetic resonance imaging (HR-MRI) has emerged as a valuable non-invasive vessel-wall imaging technique that can characterize plaque activity, including enhancement and intraplaque hemorrhage (4). Statins, widely used for atherosclerotic disease, exert lipid-lowering and pleiotropic effects (anti-inflammatory actions and plaque stabilization). While benefits are well established in coronary and carotid disease (5, 6), data remain limited regarding the impact of prior statin therapy in symptomatic ICAS. Furthermore, it is unclear whether and how pre-stroke statin use modulates specific instability features of the culprit plaque as visualized by HR-MRI. This study used HR-MRI to explore clinical factors related to unstable plaques in symptomatic ICAS and to analyze how pre-stroke statin use correlates with instability features of the culprit plaque.

## 2 Subjects and methods

### 2.1 Study subjects

We retrospectively and consecutively enrolled 116 patients admitted to the Department of Neurology, Hebei General Hospital, from January 2021 to August 2022 for acute ischemic stroke who completed HR-MRI during hospitalization.

**Figure 1:**
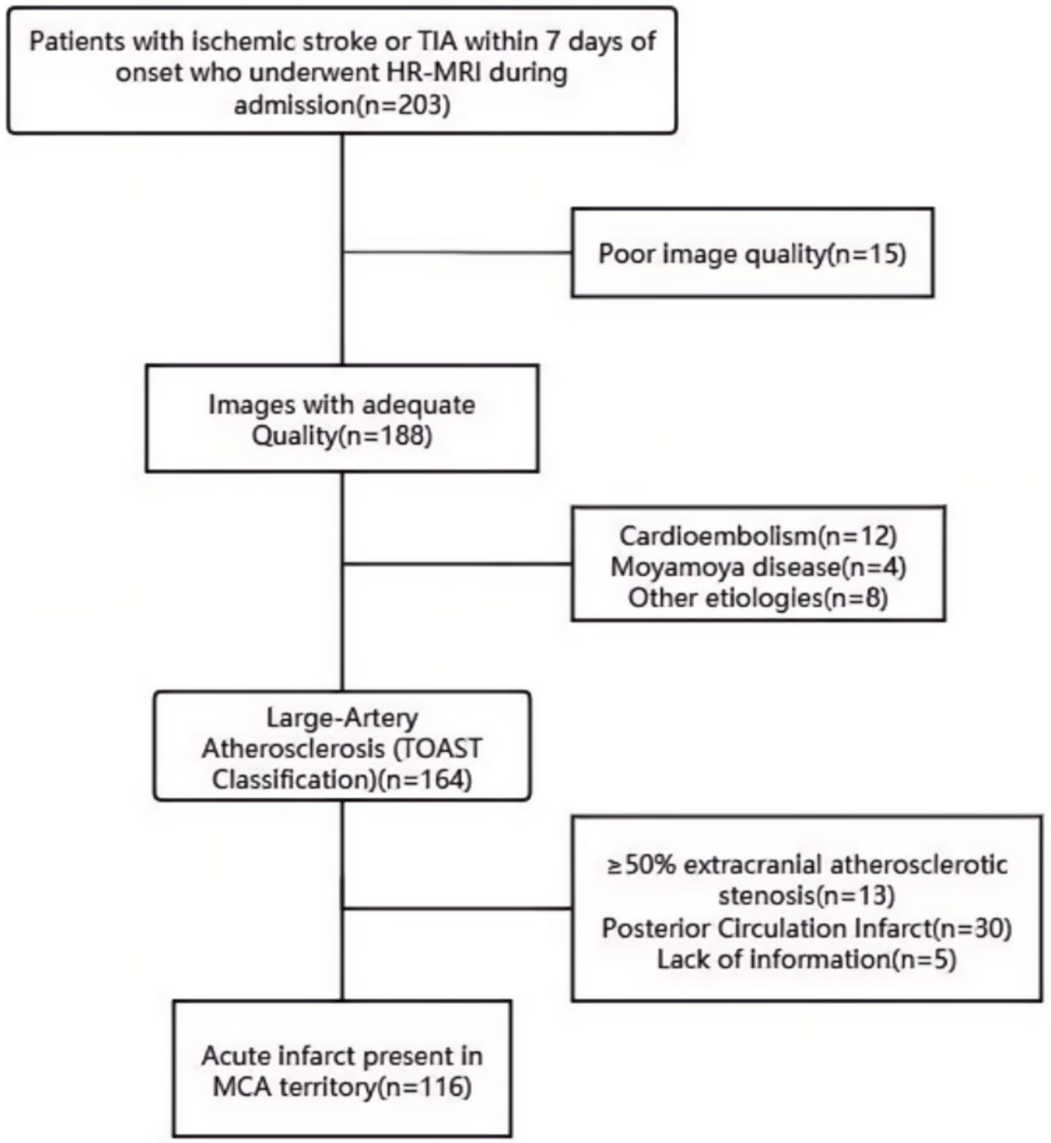
Patient selection. TIA, transient ischemic attack; HR-MRI, high-resolution magnetic resonance imaging; MCA, middle cerebral artery.

Inclusion criteria were: (1) acute (within 7 days) ischemic stroke or TIA; (2) HR-MRI completed during admission; (3) diffusion-weighted imaging (DWI) showing an acute lesion within the middle cerebral artery (MCA) territory; and (4) after systematic evaluation, large-artery atherosclerosis was deemed the most likely stroke mechanism according to a modified TOAST algorithm (7). Key modifications included: requiring HR-MRI-identified culprit plaque corresponding to the DWI lesion, excluding cases with competing embolic sources, and reclassifying small subcortical infarcts as large-artery atherosclerosis if HR-MRI showed a plaque at the relevant perforator origin. Exclusion criteria were: (1) first DWI obtained only after recanalization therapy (thrombolysis or mechanical thrombectomy); (2) ≥50% extracranial atherosclerotic stenosis in the relevant artery; (3) non-atherosclerotic vasculopathies (e.g., cardioembolism, aortic dissection, vasospasm, vasculitis, moyamoya disease, coagulopathies); and (4) suboptimal imaging quality. Patient selection is shown in Figure 1.

### 2.2 Data collection

Baseline data included sex, age, BMI (kg/m^2^), smoking history, hypertension (SBP ≥ 140 mmHg, DBP ≥ 90 mmHg, or antihypertensive use), diabetes (glucose-lowering therapy or fasting plasma glucose ≥7.0 mmol/L). Type and dose of statin were recorded at admission and converted to atorvastatin equivalents based on estimated LDL-lowering potency. Pre-stroke statin use was defined as regular intake for over 1 month prior to stroke at a daily atorvastatin-equivalent dose of less than 40 mg.

**Figure 2:**
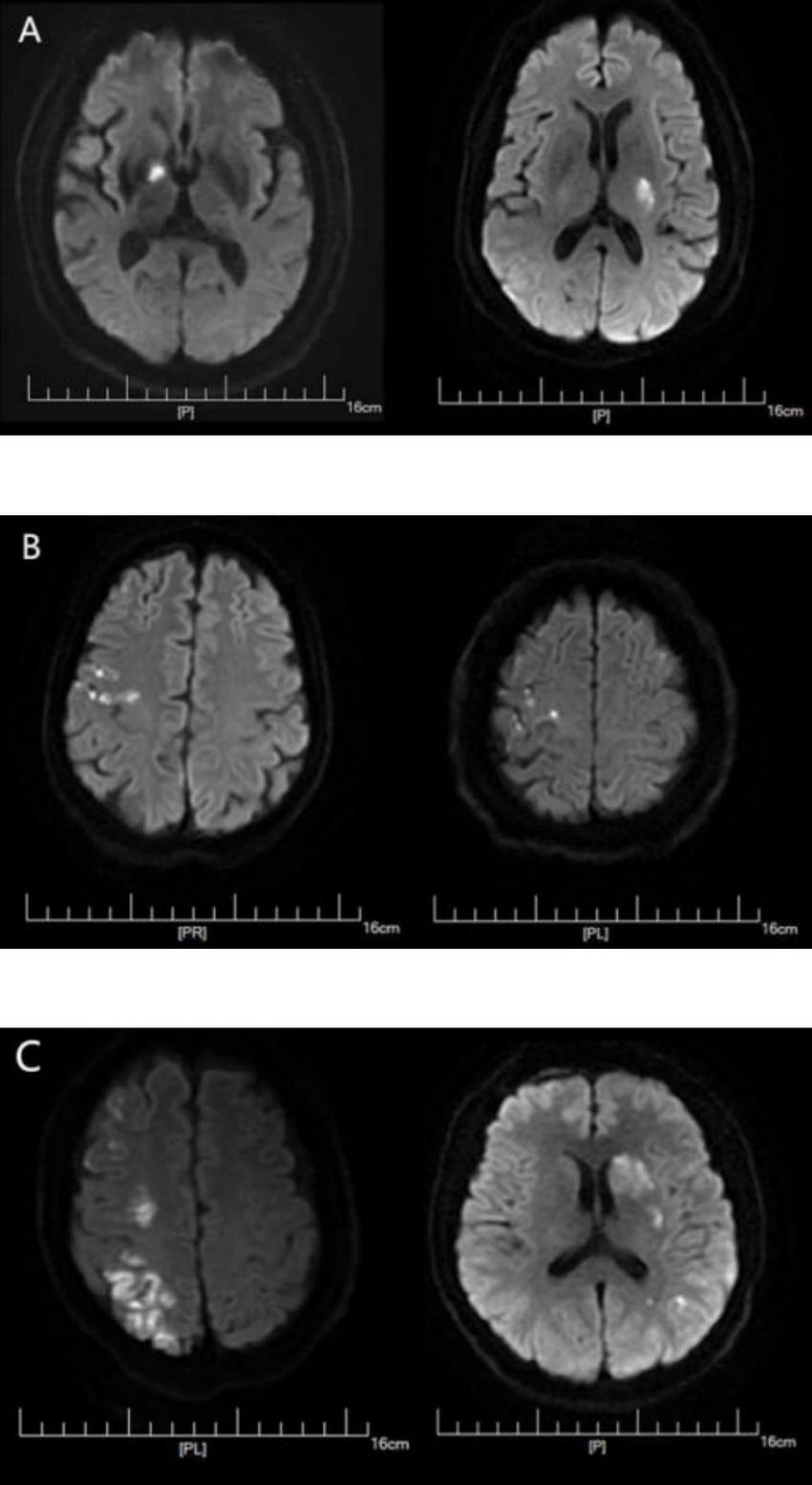
DWI lesion patterns: **(A)** deep-only pattern, **(B)** small cortical-only, **(C)** large cortical/cortical–deep pattern. The hyperintense areas in the DWI sequence images above represent the regions of acute cerebral infarction.

Admission laboratory indices included lipids (TC, triglycerides, HDL-C, LDL-C, non-HDL-C), total protein, albumin, white blood cell count, fasting glucose, and HbA1c. Lesion distribution was assessed on DWI. Patients were classified into three infarction patterns: (1) deep-only pattern—infarction confined to the striatocapsular region (basal ganglia–internal capsule), without any cortical lesion; (2) small cortical-only pattern—single or multiple cortical infarcts, each with a diameter <1 cm; and (3) large cortical/cortical–deep pattern—territorial infarction involving two or three divisions of the MCA (e.g., superior, inferior, or deep divisions) (8), as well as mixed cortical–deep infarction (9) (Figure 2).

### 2.3 MRI protocol

Imaging was performed on a 3.0-T system (Discovery MR750w 3.0T; GE Medical Systems, LLC) with a 24-channel head–neck coil. Three-dimensional time-of-flight MRA (TOF-MRA) was used to reconstruct vascular anatomy and localize stenosis. The HR-MRI protocol included coronal black-blood T1-weighted imaging (T1WI) before and after contrast. Enhancement T1-weighted imaging was performed between 1.5 and 2 min after contrast agent administration. Parameters:

1. TOF-MRA: TR 19 ms; TE 2.9 ms; NEX 1; FOV 220 × 194 mm; matrix 320 × 320; slice thickness 1.0 mm; 84 slices; the spatial resolution is 0.6875 mm × 0.60625 mm.
2. T1WI: TR 1300 ms; TE 16.7 ms; NEX 1; FOV 180 × 180 mm; matrix 320 × 320; slice thickness 0.6 mm; gap 0 mm; 124 slices; the spatial resolution is 0.5625 mm × 0.5625 mm.
3. DWI: TR 7468 ms; TE 77.2 ms; NEX 3; FOV 240 × 240 mm; matrix 130 × 160;slice thickness 4.0 mm; gap 0 mm; 72 slices; the spatial resolution is 1.85 mm × 1.5 mm. The frequency-encoding direction is left–right.

### 2.4 Image analysis

All measurements were performed on a GE Advantage workstation. Two senior radiologists independently analyzed images in a double-blinded fashion; disagreements were resolved by consensus. Interobserver agreement was excellent for plaque enhancement grading (Kappa = 0.82). Intraclass correlation coefficients for all measured high-resolution MRI parameters exceeded 0.80, indicating high consistency.

Vessels and segments for evaluation were selected based on clinical presentation. Atherosclerotic plaque was defined as eccentric wall thickening on pre-and post-contrast images, regardless of luminal stenosis (10). The culprit plaque was the sole lesion in the infarct-related arterial segment or, for multiple plaques, the most intensely enhancing plaque was selected first; if enhancement was absent or similar, priority shifted sequentially to plaques with direct high-risk features (e.g., intraplaque hemorrhage), greatest plaque burden, and finally, highest stenosis grade with closest anatomical correlation to the infarct core. At the narrowest portion (maximal plaque thickness) on coronal T1WI magnified to 300%, vessel area (VA) and lumen area (LA) were measured; corresponding measurements were obtained at a reference segment (the nearest non- or minimally diseased segment proximal to the plaque; if unavailable, the nearest distal segment was used). Wall area (WA) = VA–LA; plaque burden = WA/VA. Remodeling index (RI) = VA at the narrowest site / VA at the reference site. Stenosis (%) = (1 − LA_narrowest/LA_reference) × 100%. Plaque enhancement was graded as: grade 0—signal equal to or lower than a normal intracranial arterial wall without plaque in the same patient; grade 1—higher than grade 0 but lower than the pituitary stalk; grade 2—equal to or higher than the pituitary stalk (11).

### 2.5 Statistical analysis

Analyses were conducted in SPSS 26.0. Categorical variables are reported as counts and percentages; Continuous variables were assessed for normality using the Shapiro–Wilk test and are accordingly presented as mean ± SD or median with range. Between-group comparisons used independent-samples *t* test, one-way ANOVA, Mann–Whitney U, or Kruskal–Wallis tests (continuous variables) and *χ*^2^ test or Fisher’s exact test (categorical variables). *Post-hoc* pairwise comparisons between groups were performed with the Bonferroni adjustment/correction. Multivariable logistic regression identified independent factors associated with plaque enhancement, adjusting for potential confounders with *p* < 0.05 in univariable analyses. Odds ratios (ORs) and 95% confidence intervals (CIs) were calculated. Two-sided *p* < 0.05 indicated statistical significance.

## 3 Results

### 3.1 Clinical characteristics by pre-stroke statin use

Among 116 patients, 18 (15.5%) had taken statins before stroke onset. Baseline age, sex, BMI, hypertension, smoking, and diabetes did not differ significantly between groups. White blood cell count, fasting glucose, HbA1c, total protein, and albumin were also similar. Lipids were lower with statins: TC, LDL-C, and non-HDL-C were significantly reduced (*p* = 0.001, *p* < 0.001, *p* < 0.001), while triglycerides and HDL-C were not significantly different. Infarct-pattern distributions differed (*p* = 0.023): the statin group had more deep-only infarcts and fewer large cortical/cortical–deep infarcts; cortical-only infarcts were similar (Table 1).

**Table 1:** Clinical characteristics of patients stratified by pre-stroke statin use.

Characteristics	Nonuser (98)	Statin user (18)	*p*
Age (y)	54.8 ± 13.267	60.22 ± 11.589	0.131
Male sex [*n* (%)]	73 (84.9)	13 (15.1)	0.84
BMI (kg/m^2^)	25.976 ± 4.311	25.13 ± 3.964	0.462
Hypertension [*n* (%)]	64 (65.3)	12 (66.7)	0.911
Diabetes [*n* (%)]	23 (23.5)	8 (44.4)	0.065
Smoker [*n* (%)]	40 (40.8)	5 (27.8)	0.297
TC (mmol/L)	4.699 ± 1.195	3.646 ± 1.059	0.001*
Triglyceride (mmol/L)	1.630 ± 1.066	1.525 ± 0.906	0.787
HDL (mmol/L)	1.063 ± 0.262	0.994 ± 0.345	0. 139
LDL (mmol/L)	3.09 ± 0.819	2.231 ± 0.662	0.000*
Non HDL (mmol/L)	3.637 ± 1.019	2.697 ± 0.784	0.000*
White blood cell count (×10^9^/L)	7.588 ± 2.065	6.726 ± 1.548	0.080
Fasting glucose (mmol/L)	5.884 ± 2.340	5.547 ± 1.473	0.909
HbA1c (%)	6.582 ± 1.805	6.456 ± 1.097	0.418
Total protein (g/L)	65.563 ± 5.753	65.439 ± 4.698	0.837
Albumin (g/L)	39.347 ± 3. 161	39.456 ± 2.232	0.889
Infarct pattern			0.023*
Deep-only pattern [*n* (%)]	28 (28.6)	9 (50)	
Small cortical-only pattern [*n* (%)]	21 (21.4)	6 (33.3)	
Large cortical/cortical–deep pattern [*n* (%)]	49 (50)	3 (16.7)	

BMI, body mass index; TC, total cholesterol; HDL, high-density lipoprotein; LDL, low-density lipoprotein; HbA1c, glycated hemoglobin; Data are expressed as mean ± SD or *n* (%). *p < 0.05 was considered statistically significant.

**Figure 3:**
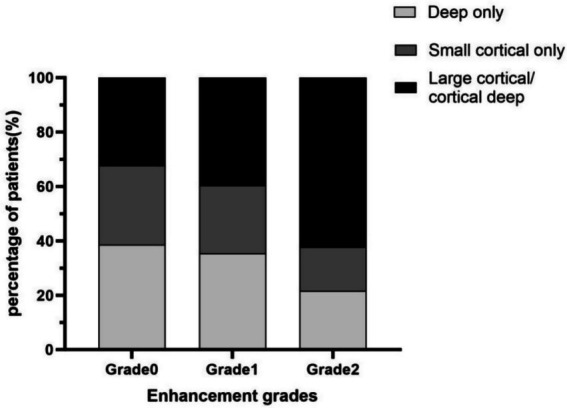
Association between culprit plaque enhancement and infarct pattern.

Associations between culprit-plaque enhancement and DWI patterns were further analyzed. Based on enhancement, 31 patients were grade 0, 48 Grade 1, and 37 Grade 2. A significant association was found between plaque enhancement and infarction patterns (*χ*^2^ = 20.274, *p* < 0.001). Bonferroni-corrected pairwise comparisons revealed a significant difference between Grade 1 and Grade 2 in basal ganglia infarcts. No significant pairwise differences were noted for cortical infarcts. For large territorial infarcts, significant differences were present between Grade 0 and Grade 2 (Table 2). Enhancement grade showed a weak positive correlation with infarct-pattern severity (Spearman’s *ρ* = 0.218, *p* = 0.019), suggesting increasing occurrence of large cortical or cortical–deep infarcts with higher enhancement (Figure 3).

**Table 2:** Comparison of infarct types by degree of enhancement.

Enhancement grades	Infarct pattern			*χ* ^2^	*p*
	Deep-only pattern [*n* (%)]	Small cortical-only pattern [*n* (%)]	Large cortical/cortical–deep pattern [*n* (%)]		
Grade 0	12 (38.71)	9 (29.03)	10 (32.26%)	20.274	0.000
Grade 1	17 (35.42)	12 (25.00)	19 (39.58%)		
Grade 2	8 (21.62)	6 (16.22)	23 (62.16%)		

### 3.2 Effects of pre-stroke statin use on HR-MRI plaque/vessel features

Between statin and no-statin groups, there were no significant differences in minimal VA, minimal LA, minimal WA, reference VA, reference LA, stenosis severity, or RI. Plaque burden tended to be lower in the statin group (*p* = 0.051). Notably, plaque enhancement occurred significantly less often with statin use (*p* = 0.015) (Table 3).

**Table 3:** HR-MRI characteristics of patients stratified by pre-stroke statin use.

Parameters	Nonuser (98)	Statin user (18)	*p*
Minimal VA (mm^2^)	14.439 ± 8.079	13.483 ± 7.09	0.711
Minimal LA (mm^2^)	4.601 ± 4. 104	5.039 ± 3.38	0.572
Minimal WA (mm^2^)	9.822 ± 6.218	8.628 ± 4.926	0.488
Reference VA (mm^2^)	18.909 ± 7.962	16.833 ± 6.20	0.301
Reference LA (mm^2^)	10.488 ± 4.387	9.911 ± 3.54	0.79
Stenosis severity (%)	57.45 ± 30.60	47.61 ± 28.969	0.223
Plaque burden	0.707 ± 0.201	0.605 ± 0. 182	0.051
RI	0.776 ± 0.293	0.824 ± 0.365	0.545
Enhancement [*n* (%)]	76 (77.6)	9 (50)	0.015*

VA, vessel area; LA, lumen area; WA, wall area; RI, remodeling index.

Data are presented as number (%) or mean ± SD. *p < 0.05 was considered statistically significant.

### 3.3 Factors associated with plaque enhancement

Univariable analysis showed significant differences between enhancement and non-enhancement groups in BMI and pre-stroke statin use (*p* = 0.016 and *p* = 0.019). Model diagnostics indicated a good fit: Hosmer–Lemeshow test *χ*^2^ = 3.009, *p* = 0.934. The discriminative validity of the model was moderate (Nagelkerke *R*^2^ = 0.137). Collinearity assessment: The VIF values for statin use and BMI were both below 10 (or 5), and their tolerance values were greater than 0.1, ruling out severe multicollinearity. In multivariable logistic regression, higher BMI (OR = 1. 157; 95% CI: 1.023–1.309; *p* = 0.021) and lack of statin therapy (OR = 3.351; 95% CI: 1.143–9.823; *p* = 0.028) were independently associated with enhancement. Age, sex, hypertension, diabetes, smoking, lipid indices (TC, triglycerides, HDL-C, LDL-C, non-HDL-C), and inflammatory/metabolic markers (white blood cell count, fasting glucose, HbA1c, total protein, albumin) did not differ significantly (Table 4).

**Table 4:** Correlates of plaque enhancement in ICAS on HR-MRI.

Characteristics	Plaque enhancement (+) (85)	Plaque enhancement (−) (31)	Univariable analysis		Multivariable analysis	
			OR (95%CI)	*p*	OR (95%CI)	*p*
Age (y)	54.8 ± 13.718	58.26 ± 11. 117	0.979 (0.948–1.012)	0.21		
Male sex [*n* (%)]	60 (70.6)	26 (83.9)	0.462 (0. 159–1.339)	0. 155		
BMI (kg/m^2^)	26.418 ± 4.216	24.277 ± 4.018	1. 161 (1.028–1.311)	0.016*	1. 157 (1.023–1.309)	0.021*
Hypertension [*n* (%)]	56 (65.9)	20 (64.5)	0.942 (0.398–2.229)	0.891		
Diabetes [*n* (%)]	22 (25.9)	9 (29)	1. 171 (0.469–2.925)	0.735		
Smoker [*n* (%)]	33 (38.8)	12 (38.7)	0.995 (0.428–2.315)	0.991		
Previous ischemic stroke [*n* (%)]	14 (16.5)	7 (22.6)	1.479 (0.534–4.096)	0.451		
Previous ischemic heart disease [*n* (%)]	7 (8.2)	3 (9.7)	1.194 (0.289–4.938)	0.807		
Antihypertensive use [*n* (%)]	51 (60)	18 (58.1)	0.923 (0.401–2.128)	0.851		
Antidiabetic use [*n* (%)]	16 (18.8)	8 (25.8)	1.5 (0.568–3.961)	0.413		
TC (mmol/L)	4.626 ± 1.277	4.287 ± 1.078	1.269 (0.888–1.815)	0. 191		
Triglyceride (mmol/L)	1.686 ± 1. 129	1.415 ± 0.721	1.387 (0.817–2.355)	0.225		
HDL (mmol/L)	1.055 ± 0.273	1.047 ± 0.289	1.099 (0.243–4.958)	0.903		
LDL (mmol/L)	3.037 ± 0.889	2.731 ± 0.715	1.554 (0.924–2.612)	0.096		
Non HDL (mmol/L)	3.571 ± 1.079	3.272 ± 0.908	1.343(0.879–2.053)	0.173		
White blood cell count (×10^9^/L)	7.536 ± 2. 141	7.230 ± 1.617	1.083 (0.873–1.343)	0.468		
Fasting glucose (mmol/L)	5.876 ± 2.411	5.71 ± 1.64	1.036 (0.853–1.258)	0.721		
HbA1c (%)	6.593 ± 1.862	6.477 ± 1.228	1.042 (0.81–1.341)	0.747		
Total protein (g/L)	65.835 ± 5.783	64.745 ± 4.997	1.037 (0.96–1. 12)	0.352		
Albumin (g/L)	39.413 ± 3. 194	39.229 ± 2.556	1.02 (0.891–1. 169)	0.771		
Pre-stroke statin use	9 (10.6)	9(29)	0.289 (0.102–0.818)	0.019*	0.298 (0.102–0.875)	0.028*

BMI, body mass index; TC, total cholesterol; HDL, high-density lipoprotein; LDL, low-density lipoprotein; HbA1c, glycated hemoglobin; data are expressed as mean ± SD or *n* (%). *p < 0.05 was considered statistically significant.

The association between intracranial arterial stenosis rate and plaque enhancement grade was analyzed using the Kruskal–Wallis test (*χ*^2^ = 27.074, *p* < 0.001). *Post hoc* pairwise comparisons were performed using the Bonferroni-corrected chi-square test. The results demonstrated statistically significant differences between Grade 0 and Grade 2, as well as between Grade 1 and Grade 2 (Table 5).

**Table 5:** Comparison of vessel stenosis rates by degree of enhancement.

Enhancement grades	*n*	*M* (*Q*_25_, *Q*_75_)	*χ* ^2^	*p*
Grade 0	31	0.37 (0.14,0.63)	27.074	<0.001
Grade 1	48	0.51 (0.23,0.74)		
Grade 2	37	0.80 (0.66,1.00)		

**Figure 4:**
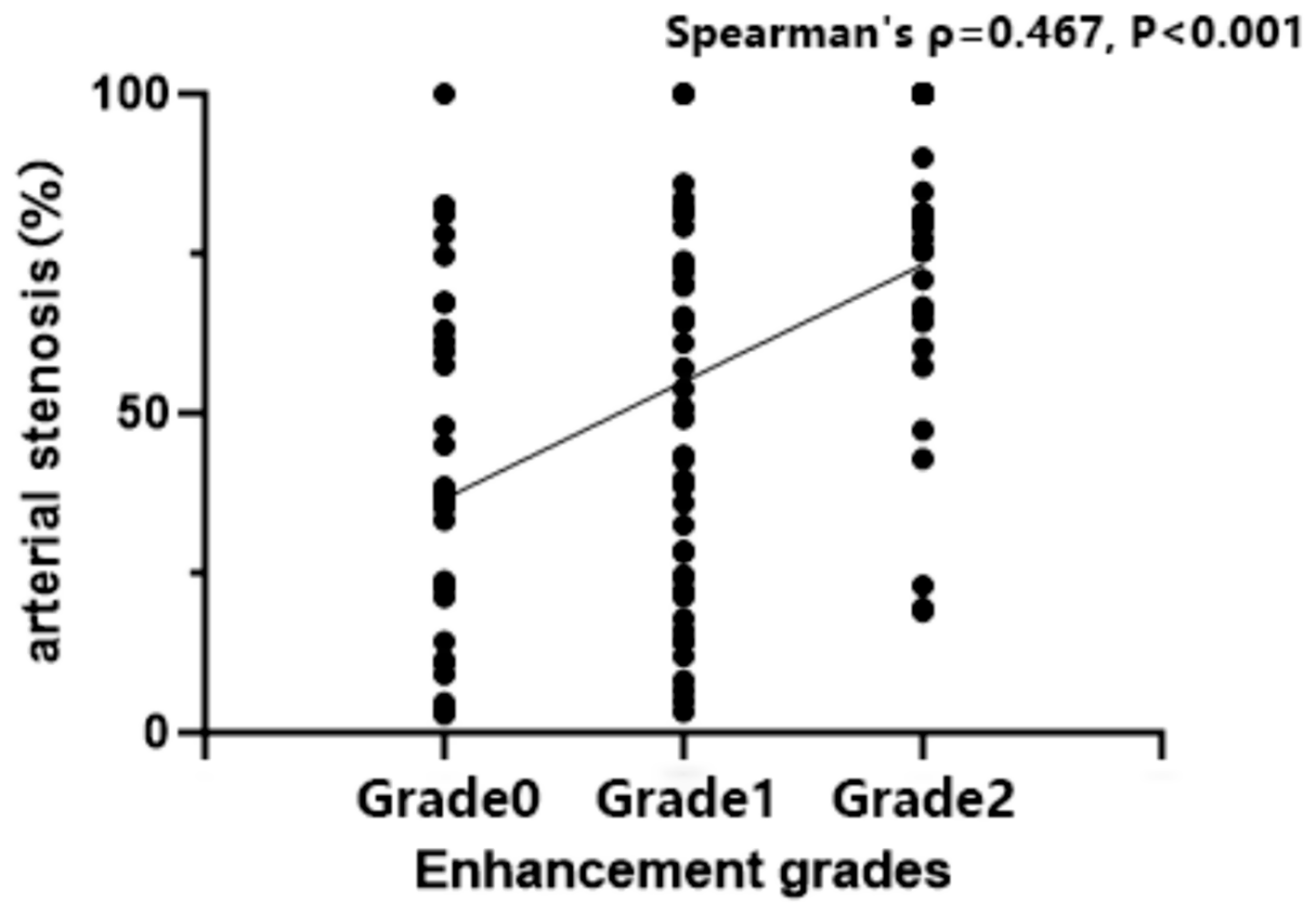
Correlation of intracranial arterial stenosis with plaque enhancement.

**Figure 5:**
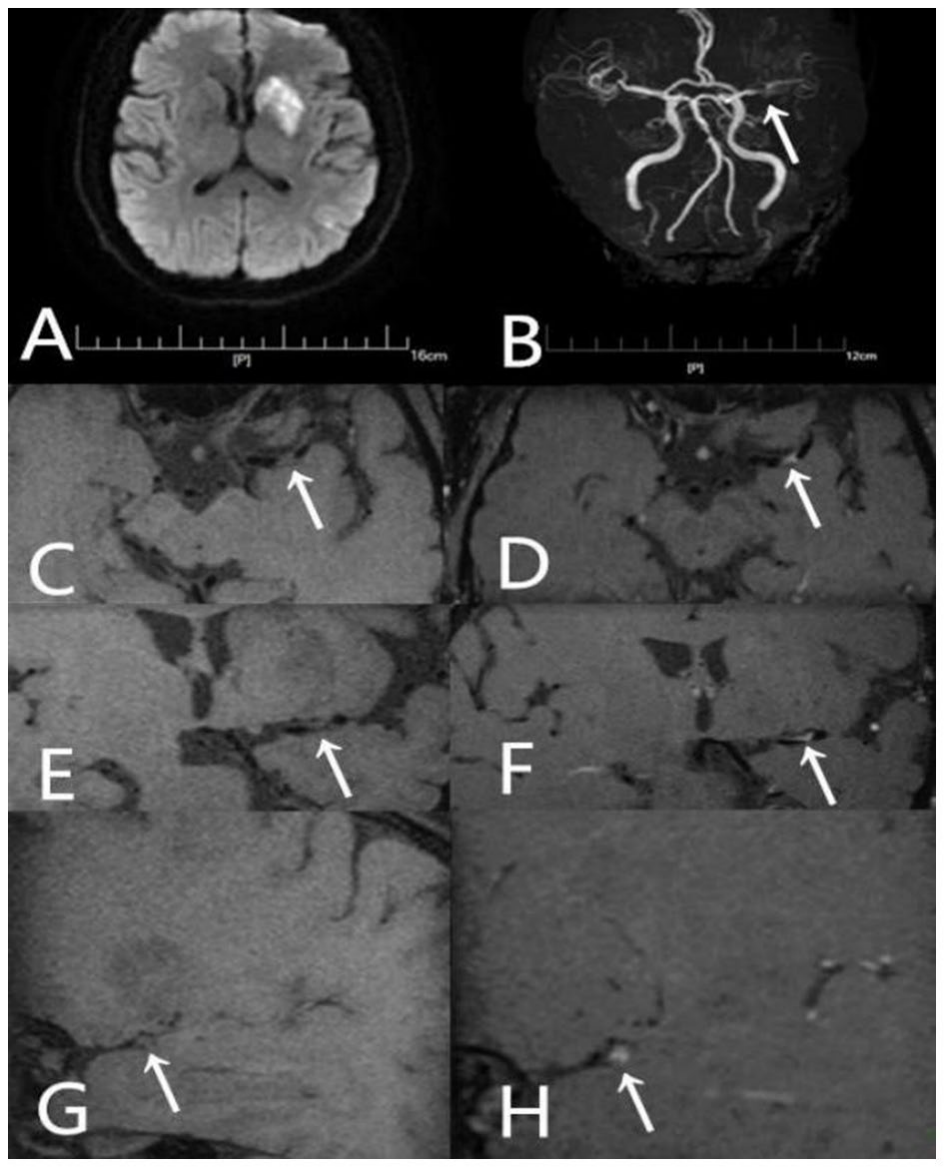
A 54-year-old male presented with aphasia and right-sided hemiparesis of one day’s duration. He had not been taking any statin medication prior to the onset. **(A)** DWI shows high signal intensity lesions in the left fronto-parieto-insular region and basal ganglia. **(B)** TOF-MRA demonstrates severe stenosis in the left middle cerebral artery (arrow). **(C)** Axial T1-weighted pre-contrast image shows the plaque (arrow). **(D)** Axial T1-weighted post-contrast image demonstrates eccentric wall thickening of the plaque (arrow) with enhancement. **(E)** Coronal T1-weighted pre-contrast image shows the plaque (arrow). **(F)** Coronal T1-weighted post-contrast image demonstrates eccentric wall thickening of the plaque (arrow) with enhancement. **(G)** Sagittal T1-weighted pre-contrast image shows the plaque (arrow). **(H)** Sagittal T1-weighted post-contrast image demonstrates eccentric wall thickening of the plaque (arrow) with enhancement.

**Figure 6:**
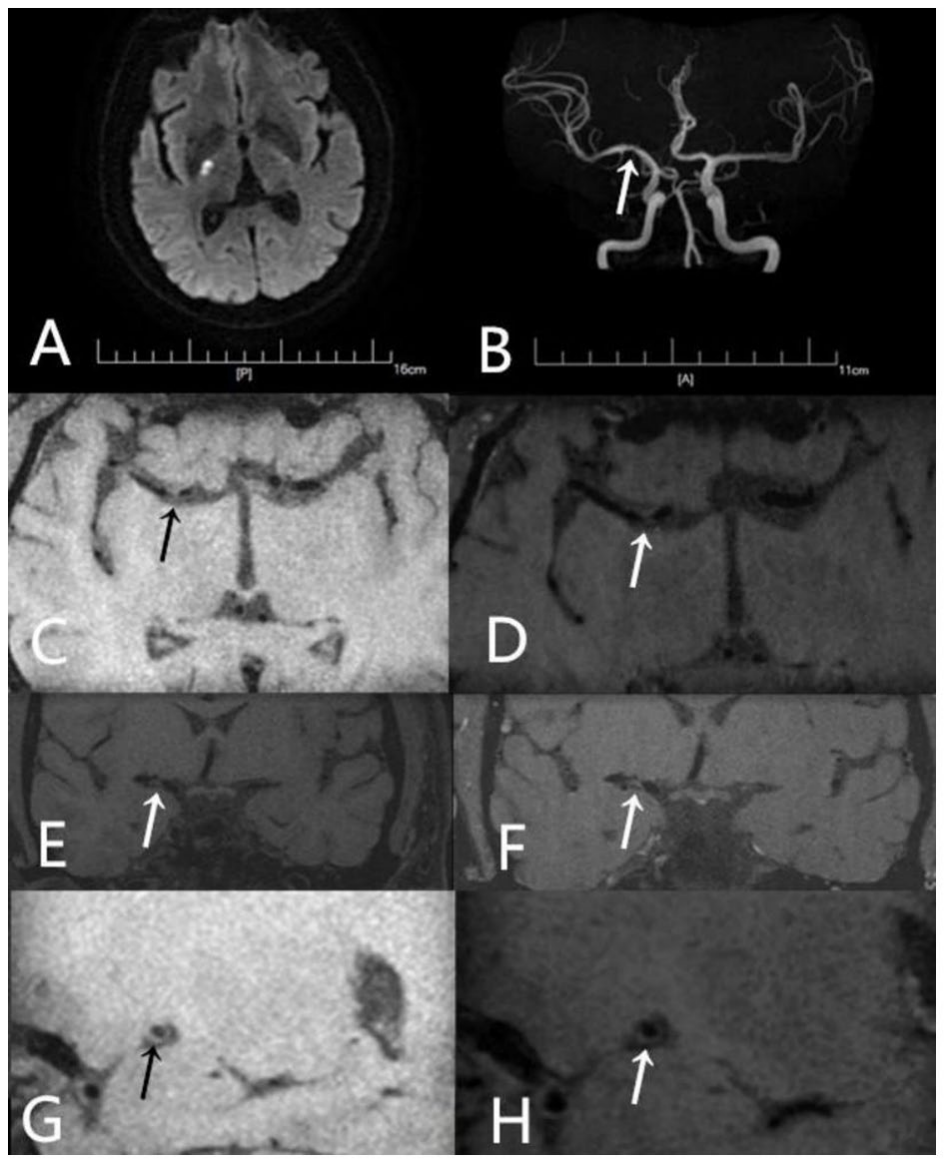
A 55-year-old male patient was admitted to the hospital due to left-sided hemiparesis for 5 days. Prior to the onset of symptoms, he had been taking Atorvastatin 20 mg once daily. **(A)** DWI shows a high signal intensity lesion in the right basal ganglia region. **(B)** TOF-MRA shows mild stenosis in the right middle cerebral artery (arrow). **(C)** Axial T1-weighted pre-contrast images shows the plaque (arrow). **(D)** Axial T1-weighted post-contrast images demonstrates eccentric wall thickening of the plaque (arrow) without enhancement. **(E)** Coronal T1-weighted pre-contrast image shows the plaque (arrow). **(F)** Coronal T1-weighted post-contrast image demonstrates eccentric wall thickening of the plaque (arrow) without enhancement. **(G)** Sagittal T1-weighted pre-contrast image shows the plaque (arrow). **(H)** Sagittal T1-weighted post-contrast image demonstrates eccentric wall thickening of the plaque (arrow) without enhancement.

Correlation analysis showed a statistically significant positive association between the degree of intracranial arterial stenosis and the extent of plaque enhancement (Spearman’s *ρ* = 0.467, *p* < 0.001). This suggests that as stenosis worsens, plaque enhancement increases correspondingly, indicating a degree of synchrony in their evolution (Figures 4–6).

## 4 Discussion

Using HR-MRI, this study examined how pre-stroke statin therapy relates to plaque stability, imaging features, and infarct-pattern distribution in ICAS, and analyzed factors associated with plaque enhancement. Statin use prior to stroke significantly reduced TC and LDL-C and was associated with lower enhancement, reflecting improved plaque stability. The statin group showed a predominance of deep-only (small, perforator-territory) infarcts and a significantly lower incidence of large cortical/cortical–deep infarcts, along with a lower frequency of enhancement. Multivariable analysis confirmed that lower BMI and statin therapy are protective with respect to enhancement (with higher BMI and lack of statin use increasing odds of enhancement). These findings suggest that statins may stabilize intracranial plaques, reduce large embolic infarcts, and thereby favorably influence stroke patterns.

On HR-MRI, plaque enhancement generally indicates neovascularization, inflammatory cell infiltration, and increased endothelial permeability—key features of plaque vulnerability (12, 13). The positive correlation between plaque enhancement and stenosis observed in this study is consistent with the findings of Huang et al. (14), who reported that more severe luminal narrowing is often accompanied by more pronounced enhancement, reflecting a greater tendency toward inflammatory activation and microvascular proliferation under hemodynamic stress. Koppara et al. (15) further demonstrated with simultaneous ^18^F-FDG PET-MRI that regions of high enhancement closely overlap with metabolically active inflammatory foci, indicating that enhancement reflects not only a morphological correlate of stenosis but also biological activity of the lesion. Importantly, the association between enhancement grade and infarct extent suggests a potential impact on clinical outcomes: higher enhancement confers an increased risk of plaque rupture and artery-to-artery embolism, ultimately leading to more extensive cerebral infarction (16). Prospective cohort studies by Gómez-Vicente and Kim et al. (17, 18) have reported plaque enhancement as an independent predictor of stroke recurrence. This finding underscores the additive risk-stratification value of plaque enhancement beyond stenosis assessment: while stenosis reflects anatomical narrowing, enhancement reflects underlying biological activity. Importantly, plaque enhancement has been shown to be a stronger predictor of future ischemic events than stenosis alone, explaining the clinical observation that patients with moderate stenosis but pronounced enhancement have a higher stroke recurrence risk, whereas those with severe stenosis but no enhancement often remain stable. In this study, patients receiving statins exhibited a significantly lower rate of intracranial plaque enhancement, with a trend toward reduced plaque burden—findings that align with the recognized pleiotropic effects of statins (19, 20).

Beyond effectively lowering LDL-C, statins directly attenuate local plaque inflammation, reduce macrophage infiltration, and reinforce the fibrous cap, thereby improving the biological stability of plaques (21–23). Multivariable logistic regression further identified higher BMI and statin therapy as independent factors associated with plaque enhancement. Recent prospective studies likewise suggest that lower BMI and longer statin exposure are associated with milder enhancement (24, 25). In addition, Zheng et al. (26) reported that combining PCSK9 inhibitors with high-intensity statins may further improve plaque stability in intracranial atherosclerotic stenosis and mitigate luminal narrowing. However, in our cohort, indices such as stenosis severity and remodeling index did not differ significantly between statin and no-statin groups. This supports the view that the stroke-preventive benefits of statins in intracranial atherosclerosis derive primarily from plaque stabilization rather than reversal of luminal stenosis. Our data also confirmed a positive correlation between enhancement and stenosis. Prior work has suggested that prolonged high-intensity statins and/or PCSK9 inhibition may have the potential to induce regression of luminal narrowing (27, 28); this effect requires confirmation in larger prospective studies.

The distribution of infarct patterns in the statin group reflected a more favorable clinical profile: a higher proportion of small deep infarcts and a significantly lower incidence of large cortical/mixed infarcts. This distribution has important implications for prognosis. Small deep infarcts, often related to isolated perforator disease arising from local lipohyalinosis or microatheroma, typically produce more limited neurological deficits and have relatively better outcomes (29, 30). In contrast, large cortical/mixed infarcts often result from artery-to-artery embolism originating from large arteries or from major trunk occlusion (e.g., internal carotid artery, middle cerebral artery), leading to extensive territorial ischemia and more severe, widespread neurological impairment with poorer prognosis (29, 31). The difference in infarct-pattern distribution associated with statin therapy is likely related to its combined effects on plaque stabilization, anti-inflammatory activity, and antithrombotic properties. By lowering lipids and reducing plaque enhancement—which reflects attenuation of intraplaque inflammatory activity and improved fibrous-cap stability—statins may reduce the risk of plaque rupture and downstream embolic events (24, 32, 33), thereby decreasing the occurrence of large cortical/mixed infarcts. By comparison, statins may have limited direct effects on isolated perforator-territory lacunes driven by local small-vessel pathology.

This study has several limitations. First, it employed a cross-sectional design with a relatively modest sample size from a single stroke center. Second, although statins are increasingly used for primary prevention, the proportion of patients with intracranial atherosclerotic stenosis (ICAS) receiving statins in this cohort remained relatively low. Patients who were on statins before their stroke likely differed systematically from non-users in terms of baseline cardiovascular risk profiles, underlying plaque biology, and engagement with healthcare services. Although our statistical adjustments controlled for several measured confounders, they may not have fully captured these baseline differences, which reduced statistical power; moreover, the limited number of statin-treated patients precluded dose-stratified subgroup analyses. Third, the timing of diffusion-weighted imaging (DWI) and HR-MRI was not standardized. Prior studies indicate that ischemic lesions on DWI and plaque enhancement on HR-MRI may evolve dynamically over the disease course (34, 35); timing variability may therefore have influenced imaging assessments. Fourth, this study lacks prospective validation and clinical endpoint correlation. The absence of long-term, prospective outcome data limits our ability to translate the observed imaging associations into validated prognostic tools. We plan to address these limitations in future, more systematic investigations.

From the imaging perspective of infarct-pattern distribution, our findings provide additional evidence for the mechanisms by which statins improve outcomes after acute ischemic stroke. Statin therapy may modulate plaque stability and inflammatory status, significantly reduce embolic events, and thereby optimize the overall pattern of post-stroke brain injury. These results reinforce the clinical value of statins in secondary prevention of ischemic stroke and underscore their pleiotropic therapeutic significance. Future studies integrating high-resolution vessel-wall imaging with long-term clinical follow-up should further elucidate the impact of statin therapy on different infarct subtypes—particularly embolic versus non-embolic stroke—to provide more precise imaging-based guidance for individualized anti-atherosclerotic treatment strategies.

## Data Availability

The raw data supporting the conclusions of this article will be made available by the authors, without undue reservation.
